# Inhaled activated protein C protects mice from ventilator-induced lung injury

**DOI:** 10.1186/cc8976

**Published:** 2010-04-19

**Authors:** Nikolaos A Maniatis, Eleftheria Letsiou, Stylianos E Orfanos, Matina Kardara, Ioanna Dimopoulou, Georgios Nakos, Marilena E Lekka, Charalambos Roussos, Apostolos Armaganidis, Anastasia Kotanidou

**Affiliations:** 1 2nd Dept. of Critical Care, "Attikon" Hospital, National and Kapodistrian University of Athens Medical School, Rimini 1, Haidari, 12462, Greece; 2 "Marianthi Simou" Laboratory, 1st Dept of Critical Care, "Evangelismos" Hospital, National and Kapodistrian University of Athens Medical School, Ipsilandou 45-47, 10675 Athens, Greece; 3 Intensive Care Department, University of Ioannina Medical School, University Campus, 45110, Ioannina, Greece; 4 Chemistry Department, University of Ioannina School of Science, University Campus, 45110, Ioannina, Greece; 5 1st Dept of Critical Care, "Evangelismos" Hospital, National and Kapodistrian University of Athens Medical School, Ipsilandou 45-47, 10675 Athens, Greece

## Abstract

**Introduction:**

Activated Protein C (APC), an endogenous anticoagulant, improves tissue microperfusion and endothelial cell survival in systemic inflammatory states such as sepsis, but intravenous administration may cause severe bleeding. We have thus addressed the role of APC delivered locally by inhalation in preventing acute lung injury from alveolar overdistention and the subsequent ventilator-induced lung injury (VILI). We also assessed the effects of APC on the activation status of Extracellular- Regulated Kinase 1/2 (ERK) pathway, which has been shown to be involved in regulating pulmonary responses to mechanical stretch.

**Methods:**

Inhaled APC (12.5 μg drotrecogin-α × 4 doses) or saline was given to tracheotomized C57/Bl6 mice starting 20 min prior to initiation of injurious mechanical ventilation with tidal volume 25 mL/Kg for 4 hours and then hourly thereafter; control groups receiving inhaled saline were ventilated with 8 mL/Kg for 30 min or 4 hr. We measured lung function (respiratory system elastance H), arterial blood gases, surrogates of vascular leak (broncho-alveolar lavage (BAL) total protein and angiotensin-converting enzyme (ACE)-activity), and parameters of inflammation (BAL neutrophils and lung tissue myeloperoxidase (MPO) activity). Morphological alterations induced by mechanical ventilation were examined in hematoxylin-eosin lung tissue sections. The activation status of ERK was probed in lung tissue homogenates by immunoblotting and in paraffin sections by immunohistochemistry. The effect of APC on ERK signaling downstream of the thrombin receptor was tested on A549 human lung epithelial cells by immunoblotting. Statistical analyses were performed using ANOVA with appropriate post-hoc testing.

**Results:**

In mice subjected to VILI without APC, we observed hypoxemia, increased respiratory system elastance and inflammation, assessed by BAL neutrophil counts and tissue MPO activity. BAL total protein levels and ACE activity were also elevated by VILI, indicating compromise of the alveolo-capillary barrier. In addition to preserving lung function, inhaled APC prevented endothelial barrier disruption and attenuated hypoxemia and the inflammatory response. Mechanistically, we found a strong activation of ERK in lung tissues by VILI, which was prevented by APC, suggestive of pathogenetic involvement of the Mitogen-Activated Kinase pathway. In cultured human lung epithelial cells challenged by thrombin, APC abrogated the activation of ERK and its downstream effector, cytosolic Phospholipase A_2_.

**Conclusions:**

Topical application of APC by inhalation may effectively reduce lung injury induced by mechanical ventilation in mice.

## Introduction

Acute lung injury (ALI), a form of pulmonary edema due to increased microvascular permeability, is a major cause of respiratory failure, morbidity and mortality in the ICU. Commonly seen as a complication of sepsis, ALI can be exacerbated by the use of mechanical ventilation, which is the main life-support modality for these patients [1,2]. Overdistention of diseased alveoli by positive pressure promotes inflammation and further disrupts the alveolo-capillary membrane [3-5]. This occurs by mechanisms including capillary stress failure, plasma membrane microrupture and lung cell death [6-9]. In addition, stretch can elicit biochemical signaling events through activation of mechanical sensors present in lung cells, including stretch-activated ion channels, the cytoskeleton, and integrins [5]. The mitogen-activated protein kinase (MAPK) enzyme family transduces many of these signals and mediates cellular responses to stretch. The importance of these enzymes is underscored by the beneficial effects observed by MAPK antagonism in various models of ventilator-induced lung injury (VILI) [10-12]. Limiting tidal volume (*Vt*) can reduce mortality by preventing ventilator injury but this complication may still occur with substantial frequency [4,13-16]. Therefore, additional therapeutic options are clearly needed and intervening in the underlying biochemical pathways with drugs could be a rational approach.

A recent development in the management of sepsis has been the launch of activated protein C (APC), an endogenous anticoagulant with additional cytoprotective, immunomodulatory and endothelial barrier-enhancing properties, which constitute important defence mechanisms in sepsis [17-21]. These attributes make APC an attractive consideration for clinical conditions associated with increased microvascular permeability in general, including ALI, irrespective of the presence of sepsis. In fact, as recently reported, intravenous APC could be useful at reversing experimental ALI caused by mechanical ventilation [22].

In clinical studies, low plasma levels of protein C have been linked to increased risk of death in patients with ALI [23] and APC supplementation improved tissue perfusion in septic patients [24]. Moreover, infusion of recombinant human APC has been shown to reduce mortality of patients with severe sepsis and at high risk of death [25-27] presumably by reversing the sepsis-associated proinflammatory and procoagulant state and thus preserving organ function. However, no benefit could be demonstrated in pediatric patients or in adults with less severe sepsis [28] or non-septic ALI [29]. These results, coupled with the drug's potential to cause bleeding and its substantial cost, have recently dampened the enthusiasm of the critical care community toward this agent and more studies have been ordered by international authorities.

In this context, exploring alternate dosing schemes and routes of administration may be helpful in elucidating how to optimally use the agent. The issue of bleeding could be addressed in the lung by local delivery, which would allow build-up of high concentrations of APC in the alveolar space, thus maximizing local cyto-protection, while minimizing the risk of systemic anticoagulation-related side effects. We and others have previously demonstrated the feasibility and efficacy of this approach in various experimental systems [30-32]. In this work we tested the utility of inhaled APC in a mouse model of ALI induced by high tidal volume (*HVt*) positive-pressure mechanical ventilation. As VILI arises from excessive stretch primarily of pulmonary epithelial and endothelial cells, we hypothesized that airspace delivery of APC would protect mouse lungs against this type of insult by targeting the affected cell populations directly. In this respect we measured lung function, indices of microvascular permeability and inflammation in order to quantify the extent of lung injury and the effect of APC. We then specifically explored the activation status of the MAPK pathway in order to gain mechanistic insight into the APC action in our model. We found that inhaled APC was associated with improved lung function and oxygenation, in addition to a reduction in lung inflammation and vascular leak. Extracellular-regulated kinase 1/2 (ERK) activation was attenuated by APC in lung homogenates, in addition to A549 human lung epithelial cells challenged with thrombin.

## Materials and methods

### Mice

Ten to fourteen week-old C57/Black6 male mice, with a mean ± standard deviation weight at the time of experiments of 22.7 ± 1.5 g, were purchased from the Biomedical Sciences Research Center 'Alexander Fleming', Vari, Greece. Mice were housed at 20 to 22°C, 55 ± 5% humidity, and a 12 hour light-dark cycle; food and water were given *ad libitum*. All experimentation was approved by an internal Institutional Review Board, as well as by the veterinary service of the local governmental prefecture.

### Reagents

All reagents were purchased from Sigma (Ilioupoli, Greece) or as specified.

### Experimental VILI

#### Outline of experimental protocol

Anesthesia was induced in mice by intraperitoneal injection of 80 mg/Kg sodium pentothal. A maintenance dose of one-third of the induction dose was required in most animals. Mice were intubated with 21 G unbeveled steel cannula via tracheostomy and connected to a Flexivent rodent ventilator (Scireq, Ontario, Canada) using ambient air to provide positive-pressure ventilation with low tidal volume (*LVt*) settings: *Vt *8 mL/Kg, and respiratory rate 150 breaths per min (bpm). Positive end-expiratory pressure (PEEP) was applied with water trap connected to the expiratory limb of the ventilator circuit. After a run-in period of five minutes, APC or normal saline (NS) were administered via nebulization as described below. Twenty minutes after treatment administration, the measurement procedure was initiated. At the beginning, two deep inflations were delivered by the ventilator to standardize volume history. This was followed by a six-minute interval of *LVt *ventilation, at the end of which lung mechanics (total pulmonary input impedance Zp by forced oscillation technique) were measured by applying an eight second oscillatory flow waveform at the airway opening. The measurement procedure was repeated twice at 30-second intervals. Immediately after lung mechanics assessment, ventilator settings were adjusted depending on the experimental group to which each animal was assigned. The measurement procedure was repeated hourly as described and was followed by administration of the treatment or sacrifice of the animal at the end of the protocol. Succinylcholine 8 mg/Kg intraperitoneally was injected hourly prior to obtaining lung function measurements and after ensuring adequate levels of anesthesia by paw pinch. For a schematic representation of the experimental protocol and measurement procedure please refer to see Figure S1 in Additional file 1.

#### Sample collection

At the end of the ventilation protocol, an arterial blood sample was obtained from the left ventricle and the abdominal large vessels were severed to allow exsanguination. Bronchoalveolar lavage (BAL) was obtained by injecting and slowly withdrawing three aliquots of 1 mL of PBS each. BAL fluid was separated from cellular components by centrifugation at 800 *g *for five minutes at 4°C and stored at -80°C. The blood was flushed out of the lungs by injecting 10 mL of PBS in the right ventricle. The right lung was snap-frozen in liquid nitrogen. The left lung was placed in 10% formalin for 24 hours and subsequently in 70% ethanol until processing.

#### Experimental groups and VILI protocol

To induce VILI, mice were ventilated with *HVt *ventilation of *Vt *25 mL/Kg, respiratory rate 50 bpm and PEEP of 2 cmH_2_O for four hours. Mice subjected to *HVt *received either 12.5 μg of recombinant human APC (drotrecogin α; Pharmaserve-Lilly, Kifisia, Greece) dissolved in 30 μL NS (*HVt-*APC group, n = 6), or 30 μL NS without APC (*HVt*-NS group n = 6). Treatments were administered as a bolus inhalation over five minutes via an Aeroneb nebulizer specially designed for use in rodents (Scireq, Ontario, Canada). In addition to the animals undergoing VILI, two control groups of *LVt *were included in this study: in the first control group, (*LVt-*30 min, n = 6) mice were ventilated with *LVt *settings for 30 minutes. Measurements in this group of animals represent values of uninjured lungs. A second control group (*LVt-*4 hr, n = 6) was ventilated with *LVt *settings for four hours, to allow assessment of the *HVt *effect during this time period (i.e. four hours) on basic parameters of lung function and inflammation (elastance, blood gases, neutrophil counts in BAL, total BAL protein levels, histology). In both *LVt *groups a PEEP of 2 cmH_2_O was applied

#### Dose-response testing

**Figure 1:**
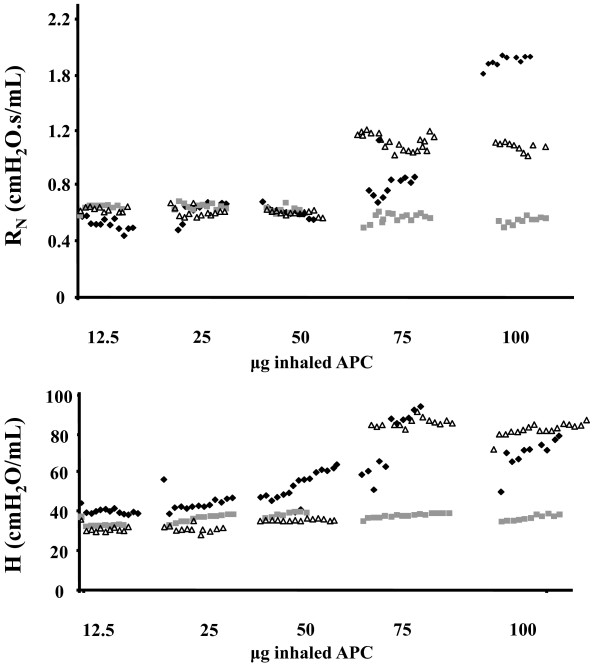
**Dose-response curve of airway resistance (R_N_) and lung tissue elastance (H) to inhaled APC**. Increasing doses ranging between 12.5 and 100 μg of inhaled activated protein C (APC) were administered to three mice and lung function was measured every 10 seconds. Airway resistance (R_N_, top) and lung elastance (H, bottom) started to increase at 25 μg. We therefore used 12.5 μg for the remaining experiments. The symbols (square, diamond and triangle) represent measurements obtained from each of the three mice used in this experiment.

Mice were ventilated with *LVt *as described. After a three-minute run-in period and two deep inflations, increasing doses of APC were administered by nebulization. A three-second forced-oscillation perturbation was applied to the airway every 10 seconds to determine lung functional properties in response to APC. Every five minutes the dose of APC was increased as shown in Figure 1, up to a maximum dose of 100 μg.

### Lung mechanics

Total respiratory system impedance Zp was measured throughout a range of frequencies applied to the airway opening during an eight-second perturbation [33]. To obtain Zp, an oscillatory wave consisting of a range of mutually prime frequencies was applied to the airway opening during an eight-second perturbation [33]. Using a mathematical model, Zp can be compartmentalized into elastance H, resistance R_N_, viscous energy dissipation G and inertance I [34]. For dose-response experiments a three-second perturbation was applied. Only values with the highest accuracy (coefficient of determination of 0.9 or greater) were included. The yield of usable measurements in this study was more than 90%.

### Arterial blood gases

Partial pressure of arterial oxygen and carbon dioxide and pH were measured using a blood gas analyzer (Radiometer Kopenhagen, Kopenhagen, Denmark)

### BAL total protein

Total protein concentration in the BAL was determined with the Bio-Rad D_c _Protein Assay kit (Bio-Rad Laboratories, Hercules, CA, USA) according to the manufacturer's instructions.

### Myeloperoxidase assay

Excised lungs were snap-frozen in liquid nitrogen, weighed and stored at -80°C. Lungs were homogenized for 30 seconds in 1 mL homogenization buffer (50 mM potassium phosphate pH 6.0, 0.5% hexadecyltrimethylammonium bromide, 5 mM EDTA) on ice. The homogenate was incubated at 60°C for two hours, centrifuged at 14,000× g for 30 minutes at 4°C and the supernatants were transferred into clean tubes. Samples of 3.75 μL were added to 296 μL of assay buffer (0.0005% H_2_O_2_, 0.167 mg/mL o-dianisidine hydrochloride in 100 mM potassium phosphate buffer) in 96-well plate in triplicates. Absorption was measured with a spectrophotometer (Tecan GENios, Männedorf, Switzerland) every 10 seconds for one minute. Thus obtained absorbance values increase linearly with time. Myeloperoxidase (MPO) activity A was expressed as change of optical density (ΔOD)/min/g of lung tissue.

### Angiotensin-converting enzyme activity

Angiotensin-converting enzyme (ACE) activity was determined in BAL fluid using fluorometric assay. Samples of 20 μL of BAL fluid were incubated with 200 μL of 5 mM Hippuryl-His-Leu for 15 hours at 37°C. The reaction was stopped with 20 μL 2.8 N NaOH and 20 μL o-pthaldialdehyde (20 mg/ml in methanol) was then added for 10 minutes at room temperature to tag His-Leu, which is released following cleavage of Hippuryl-His-Leu by ACE. Finally, 20 μL of 3N HCL were added to terminate this reaction. Fluoresence was measured at an excitation wavelength of 360 nm and emission wavelength of 465 nm (Tecan GENios, Männedorf, Switzerland). To determine ACE activity from fluorescence measurements, we used a standard curve of His-Leu.

### Western blot for ERK

Lung tissue samples were homogenized for 30 seconds in ice-cold lysis buffer (Radio-Immuno-Precipitation-Assay Buffer, 150 mM NaCl, 1% NP-40, 0.5% deoxycholic acid, 0.1% Sodium Dodecyl-Sulfate, 50 mM Tris pH = 8) Sigma (Ilioupoli, Greece) and centrifuged to separate insoluble components. Laemmli buffer was added to supernatants and samples were boiled for five minutes and subjected to electrophoresis as described [35]. Briefly, proteins were separated on a 10% SDS-PAGE and electrotransferred to polyvinylidene fluoride membranes. Membranes were blocked in buffer containing 20 mM Tris-HCl, pH 7.4, 137 mM NaCl and 0.1% Tween 20 (TBST), in addition to 5% non-fat dry milk, for one hour at room temperature. The membranes were then incubated overnight at 4°C with primary antibody (anti-phospho-Thr202/Tyr204 ERK, anti-ERK, anti-phospho Ser 505 cPLA_2 _or cPLA_2 _antibody, all Cell Signaling, Beverly, MA, USA). After washing in TBST, blots were incubated for one hour at room temperature with the appropriate horseradish peroxidase-conjugated secondary antibody. Protein bands were visualized using the Supersignal west pico chemiluminescent substrate (Pierce Biotechnology, Rockford, IL, USA). To ensure equal loading, membranes were probed with anti-tubulin or anti-actin antibody. Densitometric analysis of the films was performed with the Image J analysis software (National Institutes of Health, Bethesda, MD, USA).

### Histology and immunohistochemistry for ERK

Sections of formalin-fixed, paraffin-embedded tissue (5 μm) were deparaffinized in xylene and rehydrated in alcohol. H&E staining was performed according to standard protocols. To perform antigen retrieval for immunohistochemistry, slides were boiled in 0.01 mol/L sodium citrate buffer (pH 6.0) for 15 minutes in a microwave oven. Sections were incubated with monoclonal rabbit anti-phospho-Thr202/Tyr204 ERK (1:500) overnight and antibody binding was detected using Vectastain Elite ABC Kit (Vector Laboratories, Burlingame, CA, USA). Visualization was performed with 3,3-diaminobenzidine (Vector Laboratories, Burlingame, CA, USA) as chromogen. Slides were counterstained with H&E, dehydrated and mounted. Images were acquired with a camera mounted on an Olympus (BX50) microscope (Fotomatic AE, Athens, Greece using 40 × objective. Histological scoring was undertaken by an observer blinded to group allocation, as described [6].

### Cell culture

A549 cells purchased from American Type Culture Collection (Manassas, VA, USA) were cultured in F-12K Kaighn's medium (Gibco, Carlsbad, CA, USA) containing 10% FBS, 2 mM glutamine and 10% antibiotics. A549 cell were seeded in six-well plates and grown at 37°C, in 5% carbon dioxide, in a complete medium. Confluent cultures were incubated in serum-free medium for 24 hours. Quiescent cultures were pretreated or not with APC (10 μg/mL) for one hour and exposed to thrombin (0.5 U/mL; Enzyme Research Labs, Swansea, UK) for 15 minutes. In certain experiments, cultures were incubated with the Mitogen-Activated-Protein-Kinase-Kinase (MEK) inhibitor, UO126 (Merck KGaA, Darmstadt, Germany) dosed at 25 μM for one hour.

### Statistical analysis

**Figure 2:**
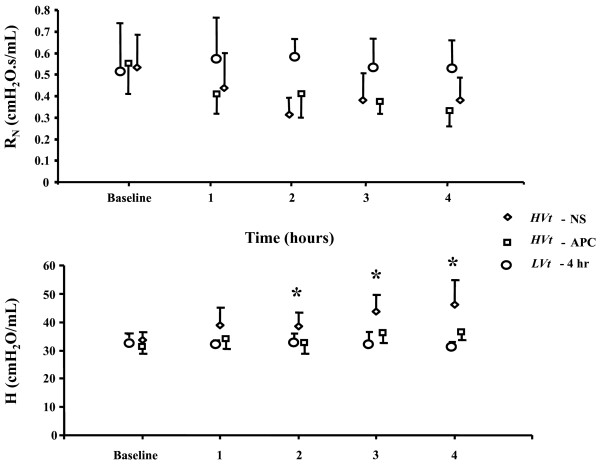
**Effect of inhaled APC on airway resistance (R_N_) and lung elastance (H) in mechanically ventilated mice**. Airway resistance and lung tissue elastance were measured hourly in mice subjected to injurious ventilation (high tidal volume (*HVt)*). Airway resistance (top) in mice of the *LVt-*4 hr group was constant throughout the experiment, whereas it trended downwards in mice receiving *HVt*. From two hours and onward, elastance (bottom) was significantly lower in mice treated with activated protein C (APC) compared with those receiving saline (NS). Mice ventilated with protective (low tidal volume (*LVt*)) ventilation served as reference (data expressed as mean ± standard deviation; * denotes *P *< 0.05; one-way analysis of variance for each time-point with *post-hoc *analysis by Dunnett's test).

Data are presented as mean ± standard deviation. Groups were compared using parametric or non-parametric one-way analysis of variance, as appropriate. For *post-ho*c comparisons, the *LVt*-30 min group was used as the reference group and all others were compared with this one using the Dunnett's test, with the exception of lung mechanics (Figure 2), where comparisons were made over time against the *LVt*-4 hr group. Groups that statistically differ from the *LVt-*30 min group are marked by an asterisk. The rationale for comparing against the *LVt-*30 min group was that this group represents the uninjured state of the lung. All *P *values are two-sided; *P *values less than 0.05 were considered statistically significant.

## Results

### Optimal inhaled APC dose

Á dose-response curve of inhaled APC was prepared by administering increasing concentrations of the drug to three mice and measuring lung elastance H and airway resistance R_N _every 10 seconds for 30 minutes (Figure 1). We found that at a dose of 25 μg there was a slight increase in H whereas R_N _increased at the 75 μg dose. We therefore used hourly inhalations of 12.5 μg/30 μL for our experiments, because this seemed to be well tolerated without changes in lung function.

### Inhaled APC prevents deterioration of lung function in response to alveolar distention

We obtained serial measurements of lung function in mice treated with inhaled APC or NS using the forced oscillation technique, an invasive but sensitive method to assess lung mechanical properties in intubated rodents (Figure 2). Baseline values of H did not differ between groups. However, after two hours of injurious ventilation and onward, H was significantly higher in mice receiving NS than in mice treated with inhaled APC or mice ventilated with *LVt*. At the end of four hours, H was 46.0 ± 8.6 cmH_2_O/mL in the *HVt-NS *group (i.e. a 36.4% increase from baseline) and 36.2 ± 2.7 cmH_2_O/mL in the *HVt-APC *group (*P *< 0.05), corresponding to a 16% increase from baseline. In mice ventilated with *LVt*, H did not increase significantly, regardless of whether NS or APC was administered (Figure 2) [see Figure S2 in Additional File 1].

### Inhaled APC preserves pulmonary microstructural integrity in VILI

**Figure 3:**
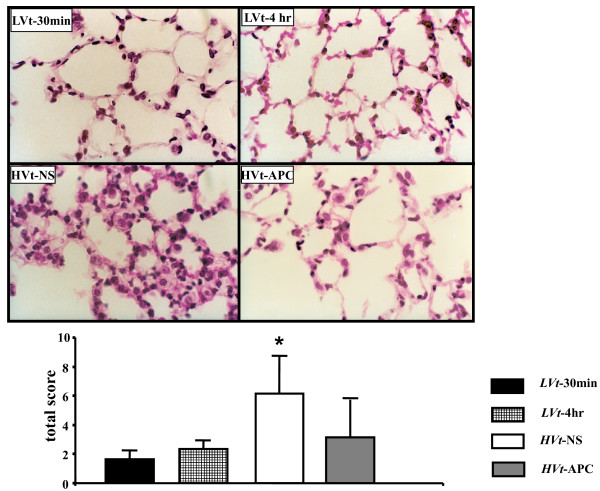
**VILI-induced microstructural alterations are attenuated by APC**. Mouse lungs ventilated with *LVt *for 30 minutes (*top left*) or four hours (*top right*) showed normal microanatomy. Application of *HVt *(*bottom left*) induced histological changes comprising in alveolar septal widening, mononuclear cell infiltration, alveolar hemorrhage and hyaline membrane formation. These changes were markedly attenuated by inhaled activated protein C (APC; *bottom right*). These observations were documented by semi-quantitative histological scoring (mean ± standard deviation; * denotes *P *< 0.05 for comparison with *LVt*-30 min; one-way analysis of variance with *post-hoc *analysis by Dunnett's test). VILI, ventilator-induced lung injury. NS, normal saline.

Examination of H&E-stained lung tissue sections (Figure 3) was performed in order to verify microanatomical features of lung injury imposed by ventilator stretch, to assess how they are affected by APC and to determine if APC administration was associated with alveolar hemorrhage. Both *LVt *groups showed normal histology (Figure 3). In the *HVt-*NS group, we found alveolar septal thickening, indicative of edema formation. We also found mononuclear cell infiltration of the alveolar walls, intraalveolar erythrocytes and hyaline membranes. These alterations were markedly attenuated in the *HVt-*APC group. No evidence of increased intraalveolar bleeding was seen in APC-treated lungs treated with *HVt *or *LVt *(Figure 3) [see Figure S2 in Additional file 1].

### Inhaled APC attenuates increased permeability response to VILI

**Figure 4:**
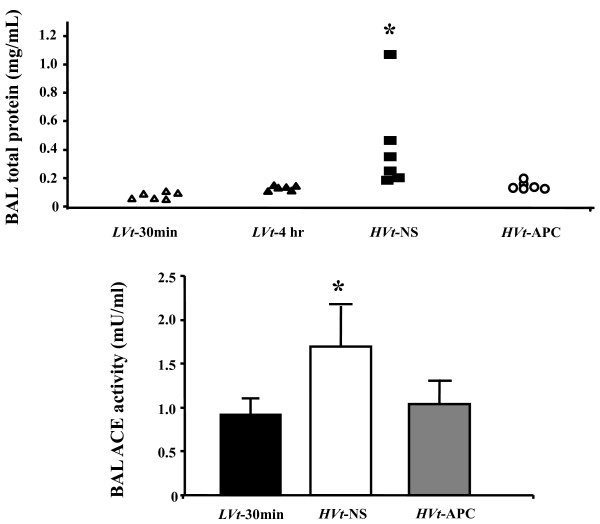
**Inhaled APC preserves alveolo-capillary barrier function in VILI**. BAL total protein concentration (top) increased in mice receiving saline (NS) and injurious ventilation (*HVt*) compared with a reference group of only briefly ventilated animals (*LVt*-30 min), indicating alveolo-capillary membrane dysfunction. Activated protein C (APC) reduced protein leak in the airspace. Changes in broncho-alveolar lavage (BAL) angiotensin-converting enzyme activity (bottom) were also reversed by APC (data expressed as mean ± standard deviation; * denotes *P *< 0.05 for comparison with *LVt*-30 min; One-way analysis of variance or Kruskal-Wallis test with *post-hoc *analysis by Dunnett's test). VILI, ventilator-induced lung injury.

To assess the integrity of the alveolo-capillary membrane, we determined total protein concentration in BAL specimens from mice undergoing control and injurious ventilation (Figure 4). Total protein increased roughly fourfold in the *HVt-*NS group compared with *LVt-*30 min, indicating lung injury due to increased microvascular permeability imposed by excessive *Vt *ventilation. In contrast, APC administration prevented the increase in permeability, indicating protection of microvascular barrier integrity by APC.

### APC prevents hypoxemia from ventilator injury

**Figure 5:**
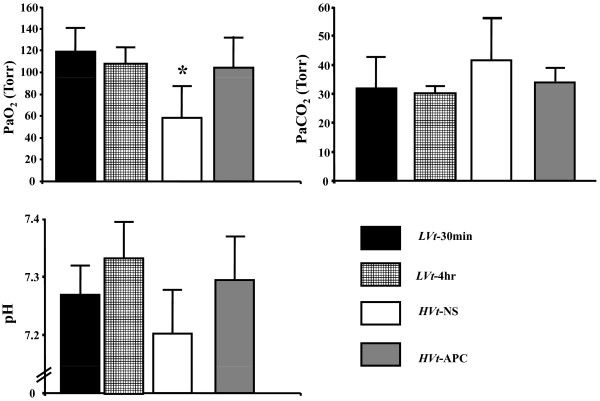
**Arterial blood gases**. Arterial hypoxemia was less in mice treated with activated protein C (APC) compared with mice receiving saline (NS). There were also non-significant trends towards acidosis and hypercarbia in the *HVt-*NS group (data expressed as mean ± standard deviation; * denotes *P *< 0.05 for comparison with *LVt*-30 min; One-way analysis of variance with *post-hoc *analysis by Dunnett's test). PaCO_2_, partial pressureof arterial carbon dioxide; PaO_2_, partial pressureof arterial oxygen.

As ALI is expected to induce hypoxemia due to ventilation-perfusion mismatching and intrapulmonary shunting, we obtained arterial blood gases at the end of each experimental protocol from our mice as markers of the above physiological alterations (Figure 5). We observed marked hypoxemia in mice subjected to *HVt *ventilation compared with both *LVt *groups (*P *< 0.05; Figure 5). In contrast, *HVt-*APC mice did not differ from *LVt *animals in blood oxygen partial pressure. In addition, we found non-significant trends towards more acidosis and hypercarbia in *HVt*-NS mice compared with *LVt *and *HVt-*APC mice (Figure 5).

### Decreased ACE activity in BAL of APC-treated mice

ACE is expressed on the surface of pulmonary microvascular endothelial cells and is shed in the bloodstream following enzymatic cleavage. In ALI, membrane-bound ACE declines and soluble ACE increases [36,37]. Thus, in the presence of lung microvascular barrier disruption, ACE may diffuse in the alveolar space. We found low levels of enzymatic ACE activity in the BAL of *LVt-*30 min mice (1.5 ± 0.3 mU/mL) but an almost twofold increase in the *HVt-*NS group (2.8 ± 0.7 mU/mL; *P *< 0.05; Figure 4). However, APC attenuated the rise in ACE activity induced by *HVt*, indicating a reduction in the degree of lung injury.

### APC-treated mice show reduced neutrophilic inflammation in airspace

**Figure 6:**
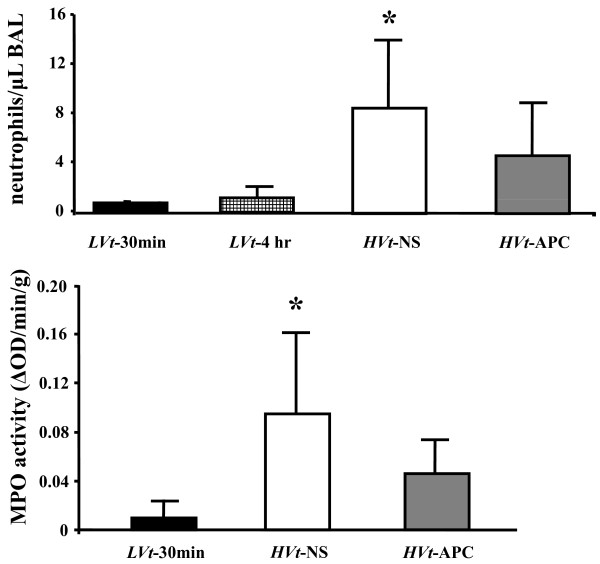
**Airspace and lung tissue neutrophils**. Broncho-alveolar lavage (BAL) neutrophil count (top) reflects airspace neutrophils. Myeloperoxidase (MPO) activity (bottom) was used to detect lung tissue neutrophil infiltration. Both inflammatory markers were reduced by activated protein C (APC), suggesting less lung injury (data expressed as mean ± standard deviation; * denotes *P *< 0.05 for comparison with *LVt*-30 min; One-way analysis of variance or Kruskal-Wallis test with *post-hoc *analysis by Dunnett's test).

As ALI, including ventilator-induced trauma, is accompanied by pulmonary inflammation, we ascertained the degree of the neutrophilic response to VILI in the air space by counting the numbers of neutrophils present in the BAL. These were markedly increased in *HVt-*NS mice compared with both *LVt *groups (*P *< 0.05; Figure 6). Administration of APC reduced BAL neutrophils by roughly 50%.

### Reduced lung tissue neutrophil infiltration by APC

We used MPO activity as a marker of lung tissue neutrophil infiltration (Figure 6). MPO activity increased in *HVt-*NS mice compared with *LVt-*30 min but to a lesser extend in the *HVt-APC *group (*P *< 0.05; Figure 6).

### Alveolar distention activates the mitogen-activated protein kinase pathway

**Figure 7:**
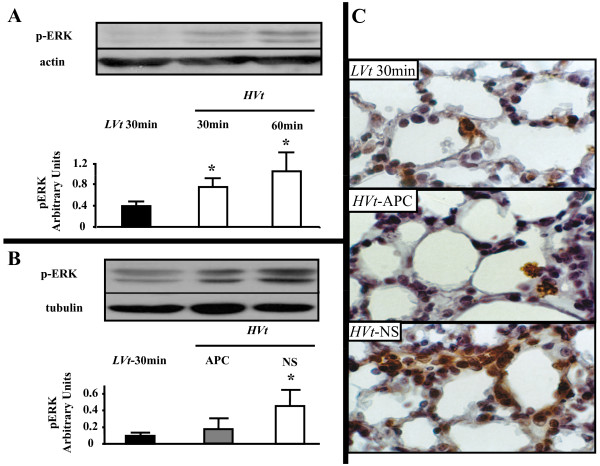
**Activation of ERK by mechanical stretch and effect of APC**. **(a) **Time-course of extracellular-regulated kinase 1/2 (ERK) activation by alveolar distention. Immunoblotting detection of phosphorylated (activated) ERK-1 (top band) and ERK-2 (bottom band) in reference to actin in mouse whole lung lysates following ventilation with tidal volume of 25 mL/Kg. ERK activation in lung tissue occurs rapidly, within 30 minutes and increases after 60 minutes of injurious ventilation. A representative blot of three experiments and band density analysis of cumulative results in reference to actin are presented. **(b) **Activated protein C (APC) reduces ERK activation in lung homogenate following ventilator-induced lung injury (VILI). Detection of activated ERK in mouse whole lung lysates by immunoblotting with antibodies binding specifically to phosphorylated ERK. ERK phosphorylation status after four hours of *HVt *ventilation remains higher in comparison with control mice. APC inhalation significantly diminishes ERK activation. A representative blot and band density analysis of cumulative results in reference to tubulin are presented. **(c) **Immunohistochemical staining for activated ERK in lung tissue. Phospho-ERK-positive cells stain brown. VILI induces activation of ERK in alveolar cells, which can be prevented by APC. (data expressed as mean ± standard deviation; * denotes *P *< 0.05 for comparison with *LVt*-30 min; One-way analysis of variance with *post-hoc *analysis by Dunnett's test).

The MAPK pathway, of which ERK is an important member, is crucial to the generation of the inflammatory response in resident lung cells. To assess the time course of ERK activation, we ventilated mice with *HVt *or *LVt *for various time points. Using immunoblotting in whole lung extract, we observed that this pathway is triggered early in the course of our experiment, already after 30 minutes of *HVt*, and increases further after 60 minutes of *HVt *(Figure 7).

### ERK activation is inhibited by APC

**Figure 8:**
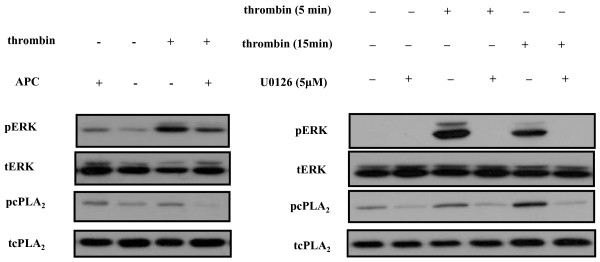
**Effect of APC on thrombin signaling pathway in epithelial cells**. **(a) **Activated protein C (APC) reverses thrombin-induced extracellular-regulated kinase 1/2 (ERK) and cytosolic Phospholipase A_2 _(cPLA_2_) activation in A549 cells. Immunoblotting detection of activated (phosphorylated) ERK and cPLA_2_. A549 cells were incubated with serum-free medium, thrombin for 15 minutes, APC for 60 minutes or APC for 60 minutes followed by thrombin for 15 minutes. APC alone induced minimal ERK and cPLA_2 _activation. In contrast, thrombin addition was associated with increased activation of ERK and cPLA_2_, both of which were abrogated in the presence of APC (Representative of five experiments). **(b) **ERK phosphorylates cPLA_2 _downstream of the thrombin receptor. ERK activation was blocked by MEK inhibitor UO126 in A549 cells challenged with thrombin. Inhibition of ERK activation led to abrogated cPLA_2 _phosphorylation (representative of three experiments).

We next examined the effect of APC administration on activation of the ERK pathway. Therefore, we determined the phosphorylation status of ERK by immunoblotting in lung tissue specimens from *HVt-*APC and *HVt-*NS mice (Figure 8). At the end of four hours, levels of activated ERK were higher in the *HVt-*NS group than in the *HVt-*APC group (Figure 8). Total ERK expression did not differ between the two groups (data not shown). In contrast to ERK, activity of c-Jun NH2-terminal kinase (JNK), another member of the MAPK family, was no different between *LVt-*30 min mice and any of the two *HVt *groups (not shown). To determine the location of activated ERK in the lung parenchyma we performed immunohistochemical staining in lung tissue sections (Figure 8). We found that phosphorylated ERK localized primarily in epithelial and endothelial cells in the alveolar septa.

### APC directly inhibits ERK activation in lung epithelial cells

To examine whether the observed effects of APC on ERK activation are the result of direct or indirect inhibition, we induced ERK activation in cultured human lung epithelial cell line A549 by thrombin. As shown in Figure 8, ERK activation by thrombin was induced within five minutes of thrombin addition to the medium and by APC alone, but to a much lesser degree. Upon pre-incubation with APC we observed that ERK phosphorylation by thrombin was markedly attenuated. We also found that phosphorylation of cytosolic phospholipase A_2 _(cPLA_2_) was also activated by thrombin but the presence of APC in the cell culture medium was associated with a reduction in phosphorylated cPLA_2_. We next examined the relation between ERK and cPLA_2 _in the presence of thrombin. To this end, cells were stimulated with thrombin in the presence and absence of MEK1 and 2 inhibitor U0126, a compound commonly used in ERK inhibition studies. We found that addition of U0126 to the cell culture medium prevented ERK activation, as expected, in addition to cPLA_2 _activation. (Figure 8).

## Discussion

In this work, we present data to show that inhaled APC ameliorates pulmonary edema and inflammation from *HVt *positive pressure mechanical ventilation. In a recent report, APC was shown to act via the endothelial protein C receptor to attenuate vascular hyperpermeability in a murine VILI model [22]; we now expand upon this observation by showing that APC via the inhalational route may also be effective in this lesion by reducing ERK activation. These two complementary animal studies support a role for APC in lung injury from excessive alveolar distention.

We and other investigators have used very large *Vt*s to cause lung injury in our studies. These ventilator settings are never encountered in clinical practice, putting into question the value of this experimental design. However, in human acute respiratory distress syndrome (ARDS) large portions of the lung may be collapsed due to edema fluid, resulting in distention of the remaining parenchyma to a much greater extent than would be expected from the *LVt*s commonly used [2]. Hence, this experimental approach may reproduce the conditions present in these relatively functional lung areas. In addition, as no other insult is present, this model allows the study of alterations directly attributed to stretch.

Initial dose-response experiments revealed that the highest bolus dose tolerated without changes in lung function is 12.5 μg in this system. Higher doses increased airway resistance and tissue elastance, possibly by inducing bronchoconstriction. Increases in airway resistance with APC have also been described with intravenous injection in rats [38], although data in humans are lacking. At the doses chosen the aforementioned phenomena were not observed and no effects on other important outcomes such as lung histology or mechanics could be documented by APC administration in lungs ventilated with protective *Vt*s [see Figure S2 in Additional file 1].

Physiological parameters, including edema and hypoxemia, were used to quantify lung injury severity in our model. Lung elastance, a measure of lung stiffness, rises progressively in rodent VILI due to such alterations as edema development and atelectasis due to surfactant dysfunction [6,39]. We have previously observed that this parameter correlates well with markers of increased vascular permeability in this model [6]. In our VILI experiments, lung function in APC-treated mice was preserved compared with mice receiving saline, which could be interpreted as protection against pulmonary edema formation by APC. Alternatively, APC may have facilitated lung edema clearance, prevented surfactant depletion or exerted an anti-inflammatory effect. In a similar respect, APC abrogated the *HVt*-induced hypoxemia, implying reductions in ventilation-perfusion mismatching and/or intrapulmonary shunting by APC. Corresponding to its effects on functional parameters, inhaled APC also prevented histological alterations induced by *HVt*. Importantly, despite the high topical dose given, no hemorrhage was observed in the lung or at any surgical site.

To assess microvascular and alveolar epithelial permeability alterations by VILI we determined BAL total protein levels and ACE activity. BAL fluid total protein rose in both *HVt *groups compared with mice ventilated with *LVt*, indicating leakage of protein-rich fluid from the intravascular to the alveolar compartment or induction of cell death. As this rise was significantly greater in saline-treated mice, APC treatment should have resulted in preservation of alveolo-capillary membrane integrity or a reduction in related cell death. To corroborate this observation using a defined plasma protein, we determined ACE activity in the BAL. Application of *HVt*s in our system led to a rise in BAL ACE activity, a phenomenon strongly attenuated by APC inhalation. Excess BAL ACE in *HVt-*NS mice likely originated from the plasma via leaky microvessels. This finding provides further evidence that APC preserves microvascular integrity in VILI.

As neutrophilic inflammation may worsen lung dysfunction in VILI, we assessed the magnitude of the pulmonary inflammatory response in our model and tested whether exogenous APC is associated with a reduction in lung neutrophil sequestration. APC administration led to a decline in both airspace and tissue neutrophils, indicating that APC may have directly affected neutrophil migration into the lung [20], and/or reduced parenchymal cell death and chemotactic cytokine production.

In order to investigate the mechanism of action of APC in our experimental model, we studied the impact of the agent on ERK activation in lung tissue. ERK is a member of the MAPK family, along with p38 and JNK. ERK is a downstream effector of small GTPase RhoA, which is activated in response to cyclic stretch and edemagenic agents such as thrombin [40]. ERK may increase permeability by promoting apoptosis, oxidant production and activation of myosin-light chain kinase, which activates molecular motor myosin to promote endothelial cytoskeletal contraction and subsequent interendothelial gap formation [12,41-43]. ERK is strongly activated in VILI experiments and has been used as an injury marker in VILI [44,45]. ERK inhibition by a small-molecule inhibitor has been effective in rescuing a mouse model of endotoxin-induced lung injury [46] and mixed models of VILI and bleomycin or hyperoxia [12,47]. In agreement with other studies [45], we found robust ERK activation within 30 minutes of *HVt *application. Importantly, ERK were still activated after four hours of *HVt *compared with *LVt*, but significantly less in APC-treated lungs. ERKactivation could be attributed directly to stretch and/or to the effects of chemical mediators, including thrombin, on alveolar cells. As APC is known to interfere with thrombin signalling pathways, particularly with regard to RhoA activation, it is conceivable that the reduction in ERK activity by APC reflects cellular, receptor-mediated effects of APC. Alternatively, APC may have led to a reduction in levels of inflammatory mediators, including activated thrombin, as has been demonstrated in bleomycin-induced fibrosis and asthma animal models of intratracheal APC administration [30,32]. In contrast to ERK, we found no difference in the activation status of JNK between groups at four hours. This indicates that blockade of ERK, due to its sustained activation, could be a useful strategy.

In *in vitro *studies we addressed the question whether the effects of APC could be attributed directly to interference with cell signaling processes as opposed to indirectly reducing levels of inflammatory mediators. We stimulated A549 human lung epithelial cells with thrombin, an important inflammatory and pro-coagulant protease with pathogenetic importance in experimental VILI [48] and found marked ERK activation, which was attenuated by APC, indicating that APC can directly alter thrombin signaling. To date, most studies have focused on the actions of APC on endothelial cells. Interestingly, however, pulmonary epithelial cells seem to express APC and thrombin receptors [49,50], even though these proteases are generally thought to be restricted to the endovascular compartment. This is one of the first reports to show that APC can modulate thrombin signaling in epithelial cells.

Activation of cPLA_2_, the rate-limiting enzyme in arachidonic acid synthesis, has been implicated in the pathogenesis of human ARDS and experimental VILI [51,52] and thrombin is an important activator of cPLA_2 _downstream of ERK [53]. However, cPLA_2 _activation can be prevented by APC. Using pharmacologic ERK inhibition in A549 cells, we show that cPLA_2 _activation by thrombin is contingent upon ERK. By preventing cPLA_2 _from accelerating arachidonic acid synthesis, APC may be exerting some of its cytoprotective and anti-inflammatory properties.

In summary, we found that topical application of APC attenuated lung dysfunction, hypoxemia, protein permeability and neutrophil infiltration in a mouse model of ventilator injury. Our study adds another piece of experimental evidence to show that APC, an endogenous anticoagulant and cytoprotective agent, could be effective in non-septic inflammatory conditions. In fact, airway application of this agent could be particularly useful to reverse lung dysfunction from mechanical ventilation. Contrary to sepsis, where patients are frequently seen at an advanced stage in the disease process, the time of onset of ventilator-associated lung trauma is well-defined, namely when the patient with ALI is placed on mechanical ventilation [54]. Thus, early administration of protective agents could prevent the severe injury incurred by artificial respiration to diseased lungs. Our investigation provides impetus to consider translational approaches addressing the role of inhaled APC in this clinical setting.

## Conclusions

In this study, we addressed the effect of airway application of inhaled APC as a prevention strategy for experimental ventilator-induced lung injury. Ventilation of healthy mouse lungs with excessive *Vt *leads to lung inflammation and edema. Inhaled APC attenuated inflammation and maintained microvascular barrier integrity, resulting in reduced lung functional impairment and hypoxemia in response to alveolar stretch. Associated biochemical events include a reduction in the activation level of ERK, providing a potential mechanism to explain the protective effects of APC administration.

## Key messages

• Airway application of APC in high doses is well-tolerated by healthy mice

• Ventilation of uninjured mouse lungs with excessive *Vt*s induces lung alterations characterized by inflammation and edema, resembling the lesions observed in the ventilated lung regions in ARDS patients

• Inhaled APC can prevent lung dysfunction in this model, and could be an interesting and relevant approach to the management of ARDS, even in the setting of coagulopathy

• ERK activation by alveolar stretch is reversed by APC, indicating that this may be a central biochemical pathway that couples mechanical signals to cellular responses

## Abbreviations

ACE: angiotensin converting enzyme; ALI: acute lung injury; APC: activated protein C; ARDS: acute respiratory distress syndrome; BAL: broncho-alveolar lavage; bpm: breaths per minute; cPLA_2_: cytosolic phospholipase A_2_; ERK: extracellular-regulated kinase1/2; FBS: fetal bovine serum; H: respiratory system elastance coefficient; H&E: hematoxylin and eosin; *HVt*: high tidal volume; JNK: c-Jun NH2-terminal kinase; *LVt*: low tidal volume; MAPK: mitogen-activated kinase; MPO: myeloperoxidase; NS: normal saline; PBS: phosphate-buffered saline; PEEP: positive end-expiratory pressure; R_N_: resistance; VILI: ventilator-induced lung injury; *Vt*: tidal volume; Zp: total pulmonary impedance.

## Competing interests

SE Orfanos has in the past received an unrestricted educational grant by Pharmaserve Lilly. The other authors declare that they have no competing interests.

## Authors' contributions

The hypothesis was developed by AK and SEO. NAM, EL and MK performed experiments. NAM performed statistics. AK, SEO, NAM and EL drafted manuscript. GN, MEL, ID, CR and AA aided with study design, data interpretation and manuscript correction. All authors have read and approved the final manuscript

## Supplementary Material

Additional file 1 **Schematic representation of experimental VILI protocol and effects of APC in mice receiving protective ventilation**. **Figure S1: Overview of experimental procedure for mechanical ventilation experiments. A**: Temporal relation of mechanical ventilation, administration of nebulized treatments (Activated Protein C-APC or Normal Saline-NS) and lung mechanics measurements. **B: **Time-course of lung mechanics measurement procedure. Deep inflation: mechanical inflation to 30 cmH_2_O. Prime-8: 8-sec forced oscillation meneuver to measure lung elastance H and airway resistance R_N _as described in 'Methods'. **Figure S2: APC administration in mice receiving protective ventilation**. We measured lung elastance H, airway resistance R_N_, BAL total protein and histological lung injury score in mice receiving nebulized NS or APC and ventilated with protective ventilation (8 mL/kg) for 4 hr as shown in Figure 1E and described in 'Methods'. We found that protective ventilation and NS inhalation induced no significant changes in lung mechanics as measured by the forced oscillation technique. In mice receiving APC we observed no significant differences in lung mechanics, total BAL protein and histological injury compared to mice receiving NS (n = 3/group). Click here for file
