# Supplementary material for: Inhaled activated protein C protects mice from ventilator-induced lung injury
Source: Crit Care. 2010 Apr 19;14(2):R70. doi: 10.1186/cc8976 (PMC2887192; doi:10.1186/cc8976)
Supplement: Additional file 1 — Schematic representation of experimental VILI protocol and effects of APC in mice receiving protective ventilation. Figure S1: Overview of experimental procedure for mechanical ventilation experiments. A: Temporal relation of mechanical ventilation, administration of nebulized treatments (Activated Protein C-APC or Normal Saline-NS) and lung mechanics measurements. B: Time-course of lung mechanics measurement procedure. Deep inflation: mechanical inflation to 30 cmH2O. Prime-8: 8-sec forced oscillation meneuver to measure lung elastance H and airway resistance RN as described in 'Methods'. Figure S2: APC administration in mice receiving protective ventilation. We measured lung elastance H, airway resistance RN, BAL total protein and histological lung injury score in mice receiving nebulized NS or APC and ventilated with protective ventilation (8 mL/kg) for 4 hr as shown in Figure 1E and described in 'Methods'. We found that protective ventilation and NS inhalation induced no significant changes in lung mechanics as measured by the forced oscillation technique. In mice receiving APC we observed no significant differences in lung mechanics, total BAL protein and histological injury compared to mice receiving NS (n = 3/group). [file cc8976-S1.DOC]

**Additional Files**

**Additional File 1: Schematic representation of experimental VILI protocol and effects of APC in mice receiving protective ventilation**

**Figure S1: Overview of experimental procedure for mechanical ventilation experiments. A**: Temporal relation of mechanical ventilation, administration of nebulized treatments (Activated Protein C-APC or Normal Saline-NS) and lung mechanics measurements. **B:** Time-course of lung mechanics measurement procedure.

Deep inflation: mechanical inflation to 30 cmH_2_O. Prime-8: 8-sec forced oscillation meneuver to measure lung elastance H and airway resistance R_N_ as described in ‘Methods’

**Figure S2: APC administration in mice receiving protective ventilation.** We measured lung elastance H, airway resistance R_N_, BAL total protein and histological lung injury score in mice receiving nebulized NS or APC and ventilated with protective ventilation (8mL/kg) for 4 hr as shown in Fig. E1 and described in ‘Methods’. We found that protective ventilation and NS inhalation induced no significant changes in lung mechanics as measured by the forced oscillation technique. In mice receiving APC we observed no significant differences in lung mechanics, total BAL protein and histological injury compared to mice receiving NS (n=3/group).
